# Prognostic significance of immune-inflammatory and nutritional indicators in locoregionally advanced nasopharyngeal carcinoma

**DOI:** 10.3389/fimmu.2026.1909926

**Published:** 2026-08-31

**Authors:** Sunqin Cai, Qingqing Chen, Jingjing Su, Ying Li, Sundong Cai, Zongwei Huang, Qunrong Cai

**Affiliations:** 1 Department of Radiation Oncology, The Second Affiliated Hospital of Fujian Medical University, Quanzhou, China; 2 Department of Otorhinolaryngology Head and Neck Surgery, The Second Affiliated Hospital of Fujian Medical University, Quanzhou, China; 3 Department of Radiation Oncology, Clinical Oncology School of Fujian Medical University, Fujian Cancer Hospital, Fuzhou, China; 4 Department of Emergency, The Second Affiliated Hospital of Fujian Medical University, Quanzhou, China

**Keywords:** immune-inflammatory index, induction chemotherapy, nasopharyngeal carcinoma, nomogram model, nutritional risk index

## Abstract

**Objective:**

This investigation was conducted to evaluate the prognostic relevance of immune-inflammatory and nutritional biomarkers in patients diagnosed with locoregionally advanced nasopharyngeal carcinoma (LA-NPC).

**Methods:**

This retrospective analysis included 267 patients. We utilized principal component analysis (PCA) for dimensionality reduction. The estimation of survival outcomes relied on Kaplan-Meier curve analysis. Multivariate Cox regression was performed to pinpoint independent prognostic factors, which facilitated the subsequent development of a nomogram. The predictive accuracy of the nomogram was evaluated using calibration curves alongside decision curve analysis (DCA). Internal validation was performed using the bootstrap resampling method with 1,000 iterations.

**Results:**

PCA yielded two major components, including C1 (immune-inflammatory index) and C2 (nutritional index). The cut-off value of C2 was -0.242. Patients with high C2 (≥ -0.242) exhibited significantly better survival than those with a low C2 (< -0.242). Multivariate analysis confirmed age, T stage, lactate dehydrogenase level, and C2 as independent predictors. The resulting nomogram demonstrated strong predictive accuracy, with a concordance index (C-index) of 0.738, and was well-calibrated. DCA indicated a greater net clinical benefit compared to conventional treatment strategies. The bias-corrected C-index was 0.7177 using the bootstrap resampling method. Using the nomogram’s risk score, patients were divided into low- and high-risk groups, with the low-risk group showing significantly superior survival. High-risk patients receiving induction chemotherapy before radiotherapy or concurrent chemoradiotherapy (RT/CCRT) experienced significantly greater gains in overall survival and locoregional relapse-free survival than RT/CCRT alone. By contrast, for low-risk individuals, equivalent efficacy was observed following either treatment approach.

**Conclusion:**

The C2 represents a reliable prognostic marker in LA-NPC. The developed nomogram enables personalized risk evaluation and aids in clinical decisions regarding tailored therapeutic approaches.

## 1 Introduction

Nasopharyngeal carcinoma (NPC) represents a distinct epidemiologic and clinicopathological entity among head and neck malignancies (1). It demonstrates a remarkable geographic disparity, with a high incidence concentrated in Southeast Asia and North Africa, reflecting a multifactorial pathogenesis that combines genetic predisposition, environmental factors, and persistent Epstein-Barr virus (EBV) infection (2). Current management, primarily centered on radiotherapy with or without concurrent chemotherapy, has significantly improved locoregional control for NPC patients (3, 4). However, a substantial proportion of patients still experience disease progression, which remains the predominant cause of treatment failure and cancer-related mortality.

Induction chemotherapy (IC) administered before definitive radiotherapy/concurrent chemoradiotherapy (RT/CCRT) offers several theoretical advantages, such as early eradication of micrometastasis and downsizing of bulky primaries to facilitate high-dose RT planning (5–7). Particularly in patients with stage N2/N3 disease or baseline plasma EBV DNA ≥ 4000 copies/mL, IC improves both locoregional control and distant-metastasis-free survival, ultimately translating into an overall survival (OS) benefit (8, 9). In contrast, IC often fails to confer survival benefits and instead increases adverse effects in low-risk NPC (10–12). The decision to administer IC should integrate the patient’s immune-inflammatory status, nutritional risk profile, physical tolerance, and comorbidities (13).

A range of hematological biomarkers have been shown to possess prognostic value across different cancer types (14, 15). Emerging evidence has established that systemic inflammation, immune dysregulation, and nutritional status exert a substantial impact on both the initiation and evolution of malignant tumors (16). Chronic inflammatory states are recognized as a trait of carcinogenesis, contributing to tumor initiation through the induction of genomic instability, promotion of angiogenesis, and remodeling of the tumor microenvironment to facilitate immune evasion (17). Additionally, nutritional assessment constitutes an essential component of NPC management, enabling individualized supportive strategies and optimizing prognostic outcomes (15, 18). Validated composite nutritional indices, including the prognostic nutritional index (PNI) and nutritional risk index (NRI), offer a more holistic evaluation by incorporating height, weight, absolute lymphocyte count, and albumin levels. Accumulating studies have demonstrated that lower pretreatment PNI and NRI values are significantly associated with worse OS, poorer response to chemoradiotherapy, and increased treatment-related toxicities in NPC patients (19, 20).

This study comprehensively evaluated the prognostic significance of immune-inflammatory and nutritional indicators for LA-NPC patients. The combination of anatomical staging and hematological biomarkers establishes a powerful basis for constructing a prognostic nomogram to predict prognosis and distinguish IC beneficiaries.

## 2 Materials and methods

### 2.1 Patient selection

This retrospective analysis consecutively enrolled 267 patients with LA-NPC diagnosed between January 2016 and October 2024. Eligible participants were required to meet the following criteria: 1) histologically confirmed stage III-IVa NPC according to the 8th edition AJCC staging system; 2) receipt of definitive intensity-modulated radiotherapy (IMRT); and 3) complete baseline laboratory data. Key exclusion criteria consisted of: 1) prior or concurrent history of other malignancies; 2) incomplete clinical records or follow-up data; and 3) severe organ dysfunction or uncontrolled systemic comorbidities.

This study was conducted in accordance with the Declaration of Helsinki and the relevant national legislation and institutional requirements, and was approved by the Institutional Ethics Committee of the Second Affiliated Hospital of Fujian Medical University (Approval No. 2025-663). Owing to the retrospective nature of the analysis, the requirement for individual informed consent was waived.

### 2.2 Data collection

Clinical characteristics and pretreatment hematological parameters were retrospectively collected from the institutional electronic medical record system. Demographic data comprised age, gender, height, weight, TNM stage, and treatment regimens. Hematological indices were obtained within one week prior to treatment initiation. Anthropometric measurements of height and weight were performed as part of routine clinical assessment. Laboratory data comprised complete blood cell counts, plasma EBV DNA levels, lactate dehydrogenase (LDH), and albumin (ALB) levels. For albumin (40–55 g/L) and LDH (120–250 U/L), the institutional reference ranges were used directly. For composite indices (platelet-to-lymphocyte ratio [PLR], monocyte-to-lymphocyte ratio [MLR], neutrophil-to-lymphocyte ratio [NLR], systemic immune-inflammatory index [SII], and systemic inflammation response index [SIRI]) and nutritional indicators (PNI and NRI), normal reference ranges were mathematically derived from institutional component normal ranges (neutrophil: 1.8-6.3; platelet: 125-350; lymphocyte: 1.1-3.2; monocyte: 0.1-0.6; all × 10^9^/L) using the standard formulas provided in the Supplementary files.

### 2.3 Treatment

The entire cohort underwent definitive IMRT as the primary radiotherapy modality. Furthermore, IC was administered to over 85% of the patients as part of their treatment regimen. For detailed methodological specifications, including chemotherapy dosing and radiation planning protocols, please refer to the Supplementary file.

### 2.4 Follow-up and outcomes

As of May 2025, the median follow-up duration was 39 months. The primary endpoint was OS, defined as the time interval from diagnosis to all-cause death. Secondary endpoints included progression-free survival (PFS), distant metastasis-free survival (DMFS), and locoregional relapse-free survival (LRRFS), measured from diagnosis to the first occurrence of disease progression (or death), distant metastasis (or death), and locoregional recurrence (or death), respectively.

### 2.5 Statistical analysis

To mitigate multicollinearity and dimensionality concerns among the variables, principal component analysis (PCA) was employed. Prior to PCA, the suitability of the data for dimensionality reduction was assessed using the Kaiser-Meyer-Olkin (KMO) measure of sampling adequacy and Bartlett’s test of sphericity. The suitability of PCA was confirmed by a KMO value exceeding 0.5 and a Bartlett’s test of sphericity yielding a P-value < 0.05. Principal components (PCs) were retained based on eigenvalues greater than 1, the scree plot inflection point, and their cumulative contribution to variance.

Survival outcomes were compared using Kaplan-Meier curves and log-rank tests. Hazard ratios (HRs) and their 95% confidence intervals (CIs) were calculated using multivariate Cox proportional hazards models. The predictive performance of the constructed nomogram was evaluated using the concordance index (C-index), calibration curves, and decision curve analysis (DCA). A two-sided P-value < 0.05 was considered statistically significant. Internal validation was performed using the bootstrap resampling method with 1,000 iterations. For each bootstrap iteration, a sample of equal size (n = 267) was drawn with replacement from the original dataset. The Cox proportional hazards model, incorporating the same predictors used in the nomogram, was fitted on each bootstrap sample. The concordance index (C-index) was calculated for both the bootstrap training sample and the original sample. The optimism for each iteration was computed as the difference between the training and test C-indices. The bias-corrected C-index was derived by subtracting the mean optimism from the apparent C-index. All analyses were performed using IBM SPSS Statistics (version 26.0) and R software (version 4.5.1).

## 3 Results

### 3.1 Baseline characteristics and survival

The study enrolled 267 patients with primary NPC. The cohort had a male predominance (male-to-female ratio: 2.3:1) with a mean age of 52.00 years. Among them, there were 152 (56.93%) and 115 (43.07%) patients with stage III and stage IVa, respectively. The majority had T3/T4 (81.27%, n=217) and N2/N3 (59.93%, n=160) classifications, and over 85% received IC. The median values of LDH, NLR, SII, SIRI, PNI and NRI were 170.00, 2.41, 686.98, 1.13, 53.95, and 111.90, respectively. The median values of all immune-inflammatory and nutritional indices fell within the normal reference ranges (**Table 1**).

**Table 1:** Baseline characteristics of nasopharyngeal carcinoma patients.

Characteristics	No. (%)/median (IQR)	Normal range reference
Age	52.00 (43.00, 59.00)	
Gender		
Female	81 (30.34%)	
Male	186 (69.66%)	
T category		
1	16 (5.99%)	
2	34 (12.73%)	
3	153 (57.30%)	
4	64 (23.97%)	
N category		
0	22 (8.24%)	
1	85 (31.84%)	
2	90 (33.71%)	
3	70 (26.22%)	
Overall stage		
3	152 (56.93%)	
4	115 (43.07%)	
IC		
No	34 (12.73%)	
Yes	233 (87.27%)	
CCRT		
No	25 (9.36%)	
Yes	233 (90.64%)	
LDH	170.00 (150.00, 199.15)	120-250
NLR	2.41 (1.83, 3.50)	0.6-5.7
PLR	156.03 (128.02, 202.54)	39-318
MLR	0.27 (0.21, 0.35)	0.03-0.55
SII	686.98 (488.96, 972.46)	70-2005
SIRI	1.13 (0.75, 1.68)	0.06-3.44
PNI	53.95 (50.98, 56.70)	45.5-71.0
NRI	111.90 (105.01, 117.19)	≥ 102
ALB	45.20 (43.00, 47.35)	40-55

IC, induction chemotherapy; CCRT, concurrent chemoradiotherapy; LDH, lactate dehydrogenase; NLR, neutrophil-to-lymphocyte ratio; PLR, platelet-to-lymphocyte ratio; MLR, monocyte-to-lymphocyte ratio; SII, systemic immune-inflammatory index; SIRI, systemic inflammation response index; PNI, prognostic nutritional index; NRI, nutritional risk index; ALB, albumin.

During follow-up, the cumulative incidence of all-cause mortality was 14.23% (n = 38). Disease progression, distant metastasis, and locoregional recurrence occurred in 21.72% (n = 58), 9.73% (n = 26), and 8.61% (n = 23) of the cohort, respectively.

### 3.2 Principal component analysis

PCA was performed on eight standardized variables representing immune-inflammatory index (NLR, PLR, MLR, SII, SIRI) and nutritional index (ALB, PNI, NRI). PCA with varimax rotation extracted two components with eigenvalues > 1, which together explained 79.4% of the total variance (**Supplementary Table 1**). The first component was termed the immune-inflammatory index (Component 1, C1), strongly associated with the immune-inflammatory biomarkers (C1; eigenvalue = 4.178, 52.2% variance), which showed strong positive loadings for NLR (0.890), PLR (0.781), MLR (0.834), SII (0.864), and SIRI (0.786), thus representing the immune-inflammatory axis. The second component was termed the nutritional index (Component 2, C2), primarily represented by the nutritional biomarkers (C2; eigenvalue = 2.175, 27.1% variance), which was characterized by high positive loadings for PNI (0.668), NRI (0.785), and ALB (0.870), representing the nutritional axis (**Table 2**). The overall KMO was 0.5591, exceeding the minimum recommended threshold of 0.5 (**Supplementary Table 2**). Bartlett’s test was highly significant (χ² = 2434.50, df = 28, *P* < 0.001; **Supplementary Table 3**). These results collectively confirmed that the correlation structure among the eight immune-inflammatory and nutritional indices was adequate for PCA.

**Table 2:** The factor loadings of each variable on the extracted components.

Variable	C1	C2
NLR	0.89	0.256
PLR	0.781	0.152
MLR	0.834	0.174
SII	0.864	0.344
SIRI	0.786	0.346
PNI	-0.658	0.668
NRI	-0.401	0.785
ALB	-0.352	0.87

C1, component 1; C2, component 2; NLR, neutrophil-to-lymphocyte ratio; PLR, platelet-to-lymphocyte ratio; MLR, monocyte-to-lymphocyte ratio; SII, systemic immune-inflammatory index; SIRI, systemic inflammation response index; PNI, prognostic nutritional index; NRI, nutritional risk index; ALB, albumin.

**Figure 1:**
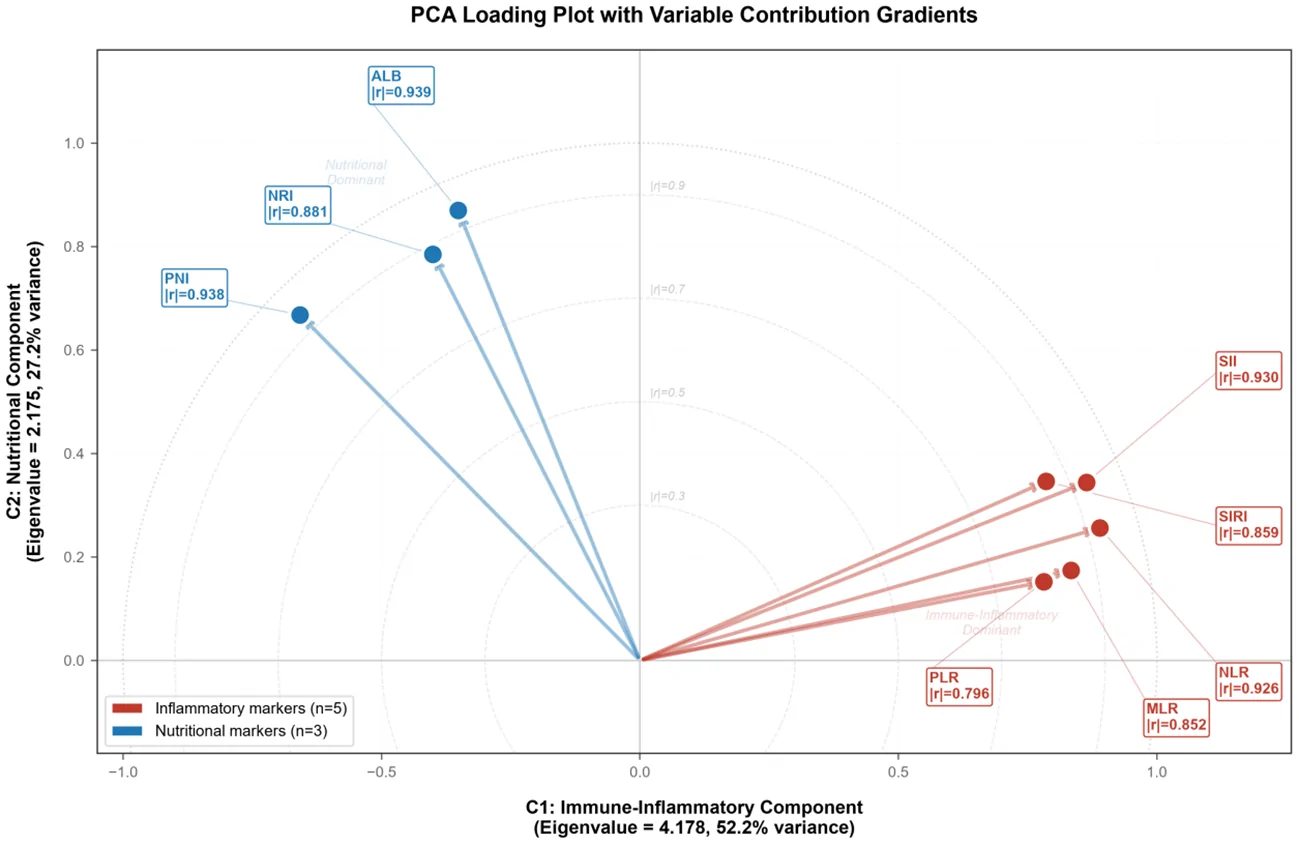
PCA loading plot with contribution gradient annotation. PCA, principal component analysis; NLR, neutrophil-to-lymphocyte ratio; PLR, platelet-to-lymphocyte ratio; MLR, monocyte-to-lymphocyte ratio; SII, systemic immune-inflammatory index; SIRI, systemic inflammation response index; PNI, prognostic nutritional index; NRI, nutritional risk index; ALB, albumin.

In the PCA loading plot with contribution gradient annotation (**Figure 1**), the eight variables exhibited a clear spatial separation along the two principal components. Each variable is represented as a vector from the origin, with the length (|r| = loading magnitude) indicating its overall contribution to the two-component model. Concentric circles at |r| = 0.3, 0.5, 0.7, and 0.9 delineate weak, moderate, strong, and very strong contribution thresholds, respectively. All five immune-inflammatory markers were positioned in the positive C1 region (loadings: 0.781-0.890), with NLR demonstrating the strongest contribution. In contrast, the three nutritional markers were located in the negative C1/positive C2 region, with ALB showing the highest loading on C2. The contribution gradient analysis revealed that all eight variables achieved loading magnitudes (|r|) exceeding 0.7, confirming the adequacy of the two-component solution in representing the original eight-variable dataset. The respective formulas for deriving C1 and C2 are provided below:

C1 = 0.435 × ZNLR + 0.382 × ZPLR + 0.408 × ZMLR + 0.423 × ZSII + 0.385 × ZSIRI - 0.322 × ZPNI - 0.196 × ZNRI - 0.172 × ZALB.

C2 = 0.174 × ZNLR + 0.103 × ZPLR + 0.118 × ZMLR + 0.233 × ZSII + 0.235 × ZSIRI + 0.453 × ZPNI + 0.522 × ZNRI + 0.590 × ZALB.

**Figure 2:**
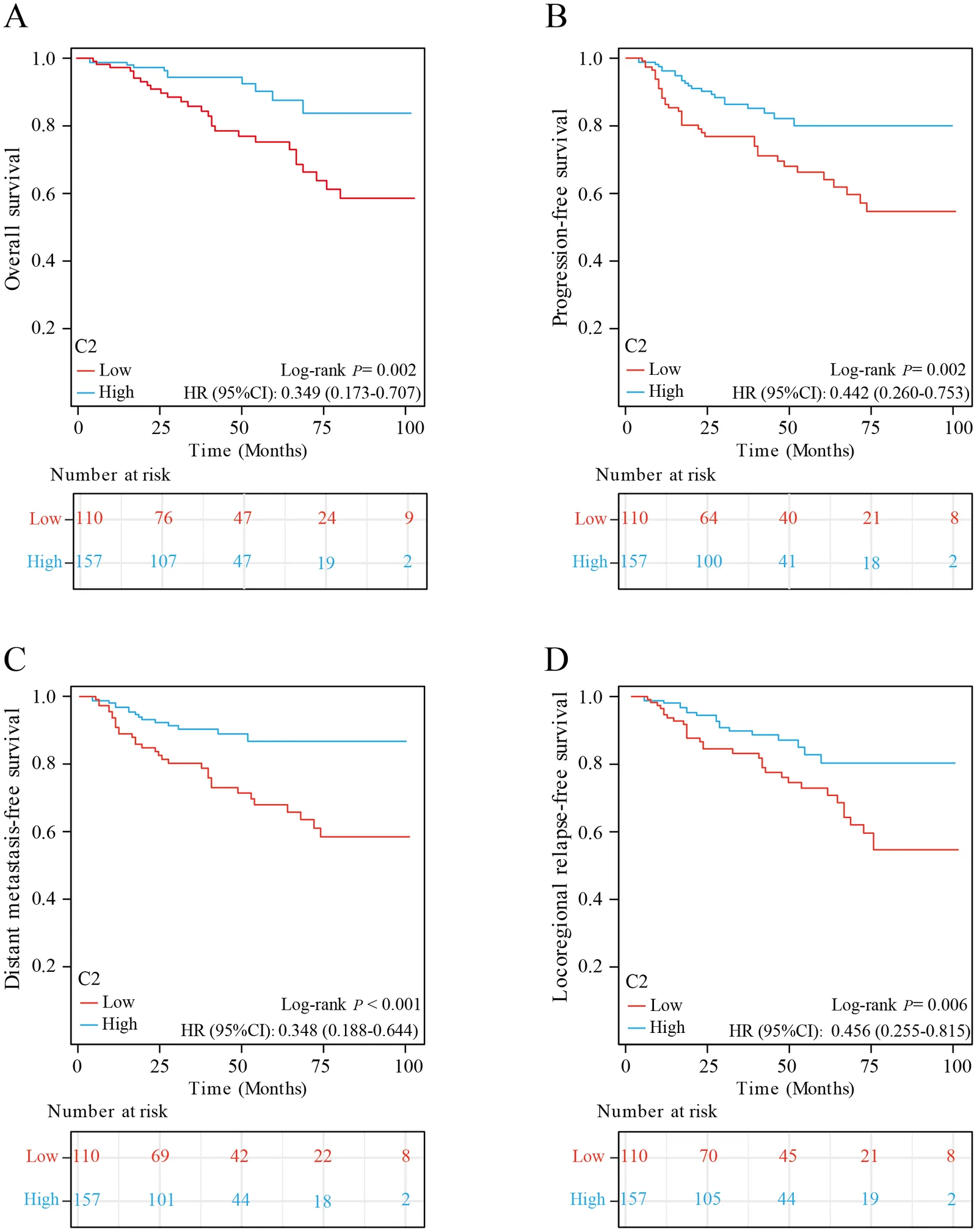
Kaplan-Meier survival curves of **(A)** OS, **(B)** PFS, **(C)** DMFS, and **(D)** LRRFS between low- and high-C2 patients. C2, component 2.

The optimal cut-off values of C1 and C2 were determined by receiver operating characteristic (ROC) curve analysis by maximizing the Youden index (sensitivity + specificity - 1) for OS, which were -0.372 and -0.242, respectively (**Supplementary Figure 1**). The corresponding area under the curve (AUC) of C2 was 0.662 (95%CI: 0.568-0.756), with sensitivity of 71.1% and specificity of 63.8%. Patients were stratified into high-C2 (C2 ≥ -0.242) and low-C2 (C2 < -0.242) groups. Subsequent survival analysis revealed significantly worse outcomes in the low-C2 group than in the high-C2 group (**Figure 2**).

### 3.3 Univariate and multivariate analyses

Multiple variables were assessed to determine potential predictors of prognosis. Univariate analysis showed that age, overall stage, C2, and LDH levels had statistically significant correlations with survival parameters (**Supplementary Table 4**). The multivariate analysis subsequently incorporated these significant variables as well as other factors of clinical relevance (**Table 3**). Multivariate analysis confirmed advanced T category (T3/T4 vs. T1/T2) as an independent predictor of poorer OS (HR: 3.40, 95%CI: 1.22-9.48, *P* = 0.020), PFS (HR: 2.77, 95%CI: 1.25-6.16, *P* = 0.012), DMFS (HR: 3.13, 95%CI: 1.25-7.80, *P* = 0.014), and LRRFS (HR: 2.80, 95%CI: 1.18-6.67, *P* = 0.020). Furthermore, elevated C2 levels independently predicted improved OS (HR: 0.38, 95%CI: 0.18-0.78, *P* = 0.009), PFS (HR: 0.47, 95%CI: 0.27-0.81, *P* = 0.006), DMFS (HR: 0.38, 95%CI: 0.20-0.72, *P* = 0.003), and LRRFS (HR: 0.48, 95%CI: 0.26-0.87, *P* = 0.015). Older age was independently associated with poorer OS and LRRFS, while LDH was an independent risk factor for OS and DMFS. C1 was not an independent factor in either univariate or multivariate analysis for any endpoint.

**Table 3:** Multivariate analysis for nasopharyngeal carcinoma patients.

	OS		PFS		DMFS		LRRFS	
Variables	HR (95%CI)	P	HR (95%CI)	P	HR (95%CI)	P	HR (95%CI)	P
Age	1.04 (1.01-1.07)	0.008**	1.02 (0.99-1.04)	0.203	1.02 (1.00-1.05)	0.097	1.03 (1.01-1.05)	0.027*
T category								
T1/T2	1.00 (Reference)		1.00 (Reference)		1.00 (Reference)		1.00 (Reference)	
T3/T4	3.40 (1.22-9.48)	0.020*	2.77 (1.25-6.16)	0.012*	3.13 (1.25-7.80)	0.014*	2.80 (1.18-6.67)	0.020*
N category								
N0/N1	1.00 (Reference)		1.00 (Reference)		1.00 (Reference)		1.00 (Reference)	
N2/N3	1.40 (0.63-3.09)	0.408	1.64 (0.86-3.11)	0.130	1.76 (0.86-3.61)	0.124	1.35 (0.68-2.67)	0.391
Overall stage								
I-II	1.00 (Reference)		1.00 (Reference)		1.00 (Reference)		1.00 (Reference)	
III-IVa	1.32 (0.66-2.63)	0.436	1.46 (0.84-2.54)	0.185	1.20 (0.64-2.23)	0.567	1.50 (0.82-2.75)	0.184
C1								
< -0.372	1.00 (Reference)		1.00 (Reference)		1.00 (Reference)		1.00 (Reference)	
≥ -0.372	0.54 (0.27-1.09)	0.088	0.77 (0.45-1.33)	0.357	0.76 (0.41-1.40)	0.377	0.62 (0.34-1.12)	0.110
C2								
< -0.242	1.00 (Reference)		1.00 (Reference)		1.00 (Reference)		1.00 (Reference)	
≥ -0.242	0.38 (0.18-0.78)	0.009**	0.47 (0.27-0.81)	0.006**	0.38 (0.20-0.72)	0.003**	0.48 (0.26-0.87)	0.015*
LDH	1.01 (1.01-1.01)	0.017*	1.00 (1.00-1.01)	0.101	1.01 (1.01-1.01)	0.020*	1.00 (1.00-1.01)	0.089

OS, overall survival; PFS, progression-free survival; DMFS, distant metastasis-free survival; LRRFS, locoregional relapse-free survival; C1, component 1; C2, component 2; LDH, lactate dehydrogenase; *P < 0.05; **P < 0.01.

### 3.4 Construction of nomogram model and bootstrap internal validation

**Figure 3:**
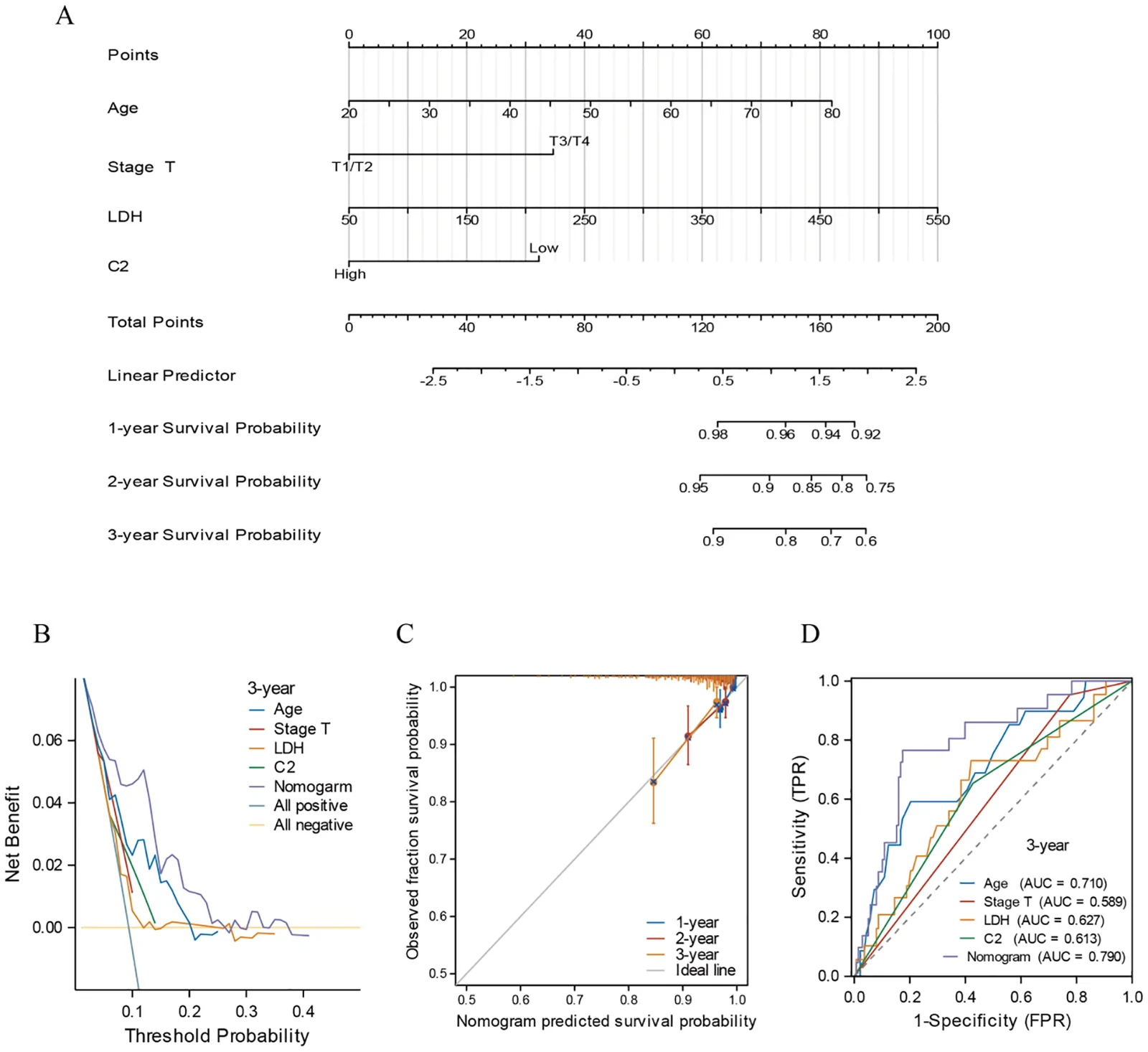
Prognostic nomogram constructed with key predictive variables **(A)**. 3-year OS decision-curve assessment **(B)**. Calibration curves for 1-, 2-, and 3-year OS **(C)**. The receiver operating characteristics curve for 3-year OS **(D)**. OS, overall survival; ROC, receiver operating characteristic; C2, component 2; LDH, lactate dehydrogenase.

To predict OS, a comprehensive prognostic nomogram was constructed by incorporating independent predictive variables: age, T stage, LDH level, and C2 group. This tool provides individualized probability estimates for 1-, 2-, and 3-year OS (**Figure 3A**). The model exhibited good discrimination, yielding a concordance index of 0.738 (95%CI: 0.696-0.780). Calibration analysis revealed strong consistency between the predicted and observed survival outcomes (**Figure 3B**). The DCA additionally indicated that compared with standalone prognostic variables, the nomogram delivered enhanced net clinical benefit over an extensive range of threshold probabilities (**Figure 3C**). The AUC of ROC for 3-year OS prediction was 0.790 (**Figure 3D**). Collectively, these observations verify that the nomogram accurately predicts survival in patients with LA-NPC.

Internal validation was performed using the bootstrap resampling method with 1,000 iterations (**Supplementary Figure 2**; **Table 4**). The bias-corrected C-index was 0.7177 (95%CI: 0.640-0.804), which is slightly lower than the apparent C-index of 0.7380 after correcting for an optimism of 0.0203. The narrow confidence interval and the small optimism (approximately 2.7% of the apparent C-index) indicate that the nomogram’s predictive performance is robust and not substantially overfitted. The model demonstrates acceptable discriminative ability with a bias-corrected C-index well above 0.5, confirming that the combination of age, T stage, LDH, and C2 provides meaningful prognostic stratification for OS in LA-NPC patients.

**Table 4:** Bootstrap internal validation results.

Parameter	Value
Total patients	267
Events (deaths)	38 (14.2%)
Bootstrap iterations	1,000
Apparent C-index	0.7380
Mean optimism	0.0203
Optimism standard deviation	0.0422
Bias-corrected C-index	0.7177
95% CI (percentile method)	0.640-0.804

### 3.5 Risk stratification and subgroup analysis

**Figure 4:**
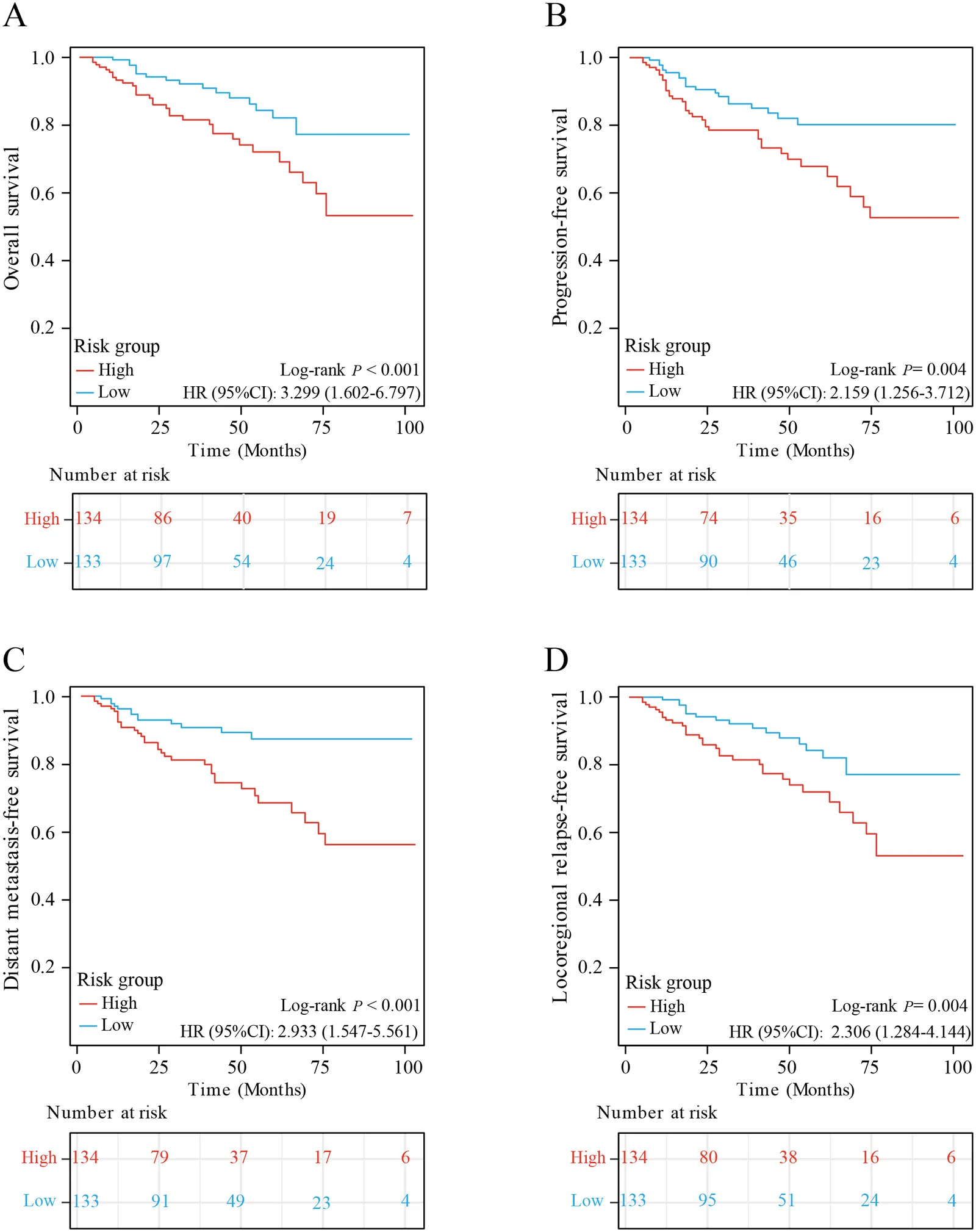
Kaplan-Meier survival curves of **(A)** OS, **(B)** PFS, **(C)** DMFS and **(D)** LRRFS for NPC patients in different risk groups.

Using risk scores calculated from the nomogram, patients were classified into high- and low-risk cohorts based on the median value of 109.4. Subsequent survival analysis indicated that the low-risk group had markedly superior survival relative to the high-risk counterparts, with 3-year OS (97.4% vs. 83.6%, *P* < 0.001), PFS (85.9% vs. 77.9%, *P* = 0.004), DMFS (90.6% vs. 80.9%, *P* < 0.001), LRRFS (92.0% vs. 81.2%, *P* = 0.004) all favoring the former (**Figure 4**). These findings further validate the robust discriminatory power of the prognostic model.

**Figure 5:**
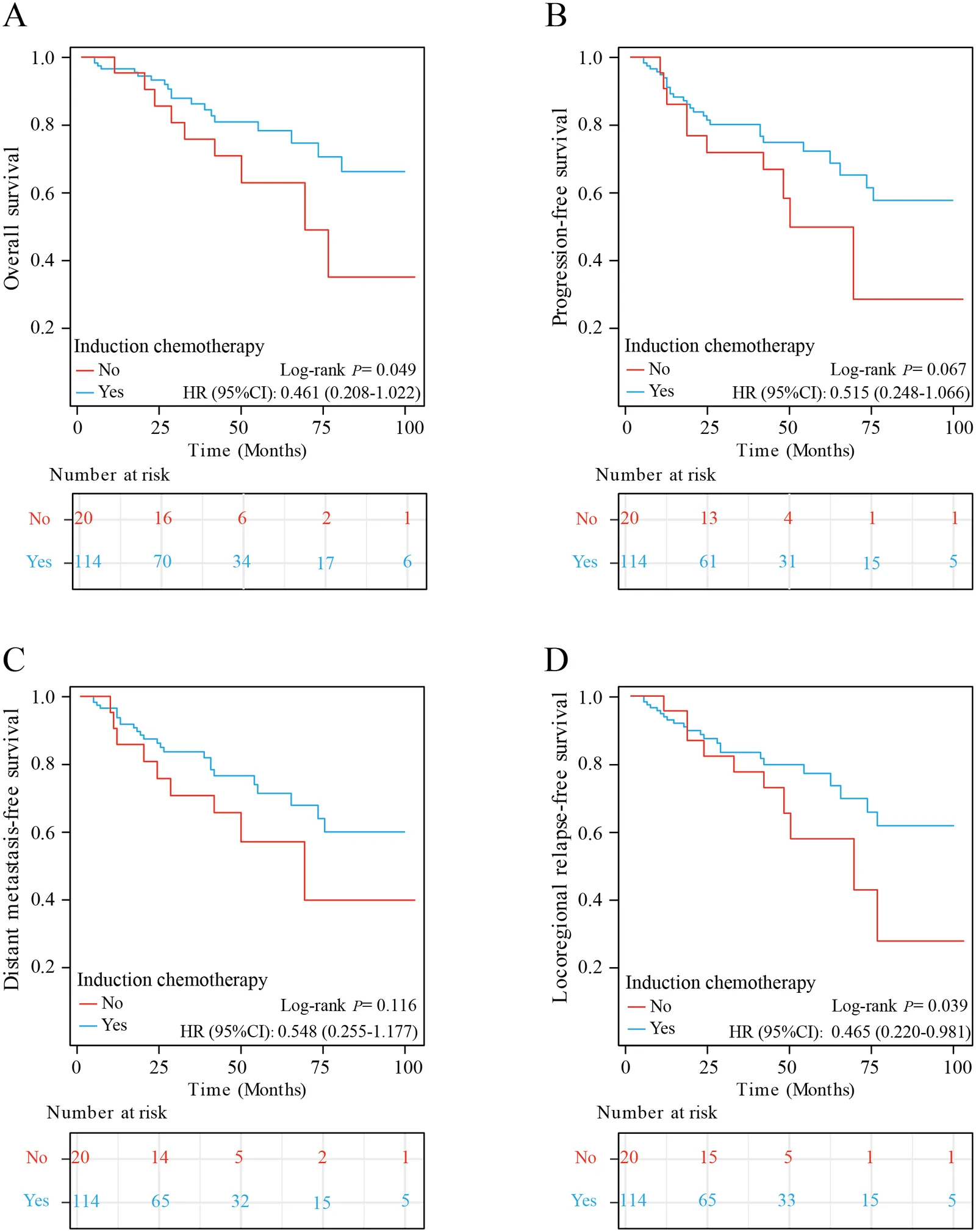
Kaplan-Meier survival curves of **(A)** OS, **(B)** PFS, **(C)** DMFS and **(D)** LRRFS for NPC patients receiving induction chemotherapy in the high-risk group.

Further risk-stratified analysis demonstrated significant differences in treatment response between the therapeutic regimens, particularly among high-risk patients. In the high-risk subset, adding IC to RT/CCRT significantly prolonged both OS and LRRFS relative to RT/CCRT alone (**Figure 5**). Conversely, low-risk patients exhibited similar survival curves under the two strategies, with no measurable benefit from intensified therapy (**Supplementary Figure 3**).

### 3.6 Dynamic C2 monitoring during chemoradiotherapy

We further explored the relationship between dynamic changes in C2 during chemoradiotherapy and the optimization of prognostic prediction. We have incorporated C2 measurements at three time points: pre-treatment C2 (pre-C2, within 1 week prior to treatment initiation), pre-radiotherapy C2 (pre-RT C2, within 1 week before RT initiation), and end-of-radiotherapy C2 (end-RT C2, within ±1 week of RT completion). All C2 values were calculated using the same formula as for pre-C2, with the identical cutoff of -0.242 applied for binary classification [high-C2 (C2 ≥ -0.242) and low-C2 (C2 < -0.242) groups].

The descriptive statistics of C2 at three time points were shown in **Supplementary Table 5**. The analysis encompasses 267 NPC patients with complete pre-C2 and pre-RT C2 data; 256 patients (11 missing) had available end-RT C2 data. Regarding the prognostic value of dynamic C2 monitoring, at the population level, C2 values do not show a systematic drift across treatment phases (all paired t-tests P > 0.5, **Supplementary Table 6**).

**Figure 6:**
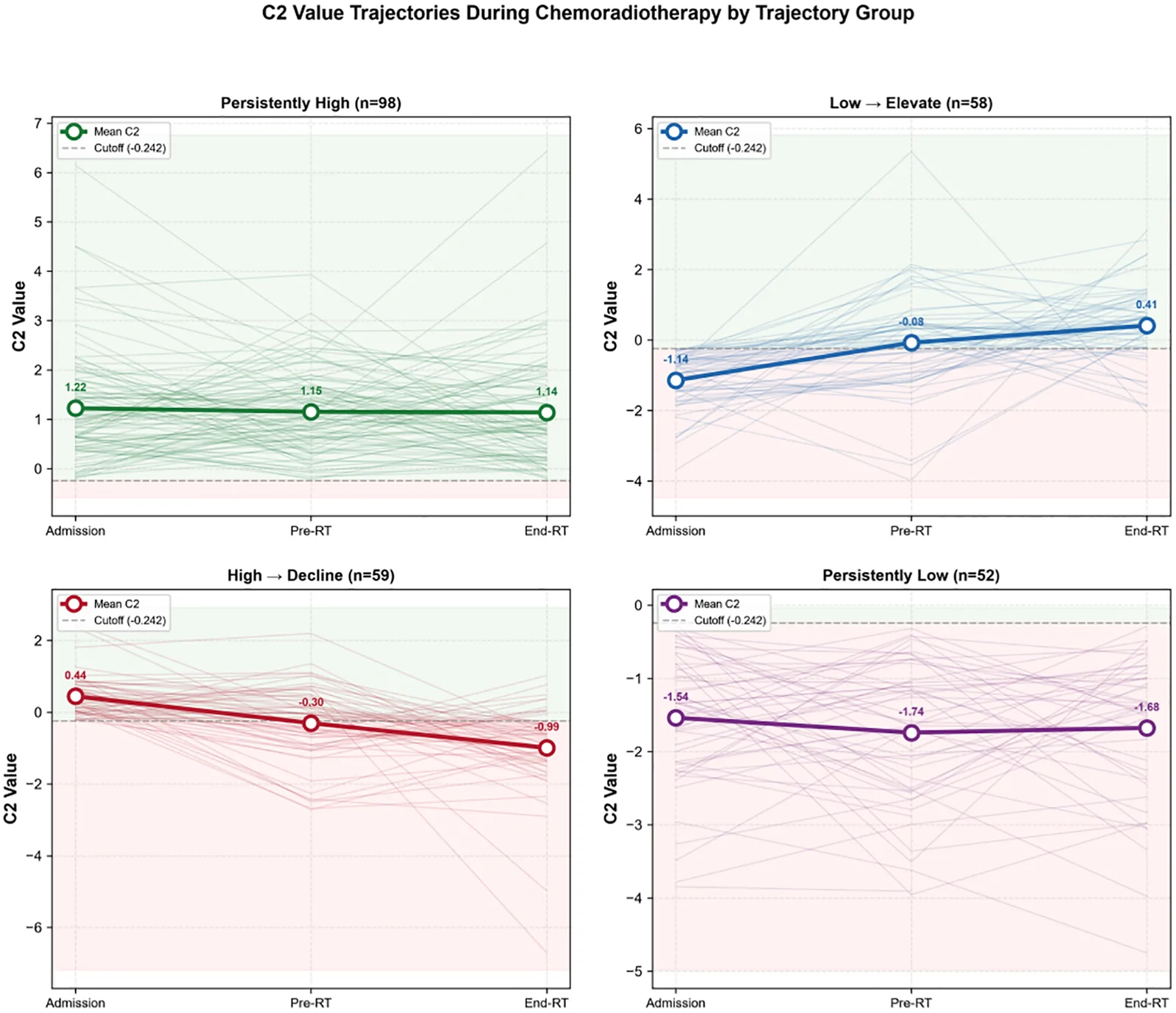
Individual C2 value trajectories across three time points by trajectory group.

**Figure 7:**
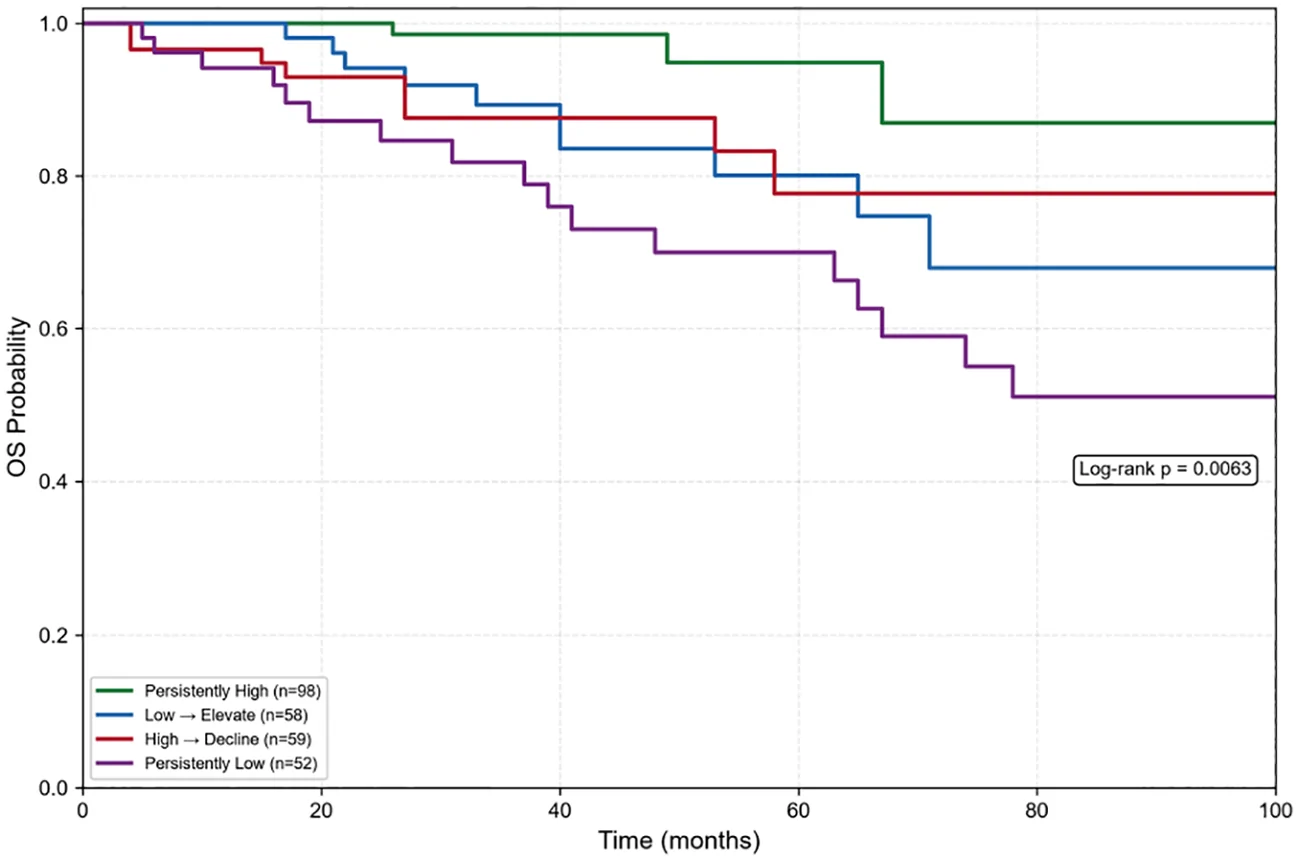
Kaplan-Meier overall survival curves stratified by C2 trajectory group.

Patients were classified into four clinically meaningful trajectory groups based on their C2 cutoff status transitions from admission to pre-RT (and end-RT when available; **Table 5**; **Figure 6**). Kaplan-Meier OS curves stratified by C2 trajectory group were shown in **Figure 7**. The persistently high group demonstrates the best prognosis (3-year OS: 98.4%), while the persistently low group has the worst (3-year OS: 84.5%). Overall log-rank test: chi-squared = 12.35, *P* = 0.0063.

**Table 5:** C2 trajectory group distribution and survival outcomes.

Trajectory group	N	%	Events	Event rate	3-year OS
Persistently High	94	35.2%	3	3.2%	98.4%
High → Decline	59	22.1%	8	13.6%	87.6%
Low → Elevate	58	21.7%	10	17.2%	89.3%
Persistently Low	46	17.2%	14	30.4%	84.5%

OS, overall survival; C2, component 2.

In the univariate analysis, pre-C2 was independently and significantly associated with OS (HR = 0.35, 95%CI: 0.17-0.71, *P* = 0.003; **Supplementary Table 7**). In the multivariable model simultaneously incorporating pre-C2, pre-RT C2 and end-RT C2, the pre-C2 retained statistical significance (HR = 0.42, 95%CI: 0.19-0.94, *P* = 0.034), and end-RT C2 was independently and significantly associated with OS (HR = 0.38, 95%CI: 0.18-0.81, *P* = 0.013), while pre-RT C2 did not (*P* = 0.530).

### 3.7 Sensitivity analysis for missing EBV DNA data

EBV DNA load was not included as a parameter in the nomogram in this study, because the EBV DNA detection technique at our center was not yet fully mature in the early stage, and the EBV DNA data in this cohort were also incomplete. To quantify the potential bias introduced by these missing data and to evaluate whether incorporating EBV DNA would alter the nomogram’s predictive performance, we conducted a comprehensive sensitivity analysis.

Among the 267 patients in our cohort, plasma EBV DNA was available for 190 (71.2%), with 103 undetectable (negative) and 87 detectable (positive). The 77 patients (28.8%) with missing EBV DNA were treated in earlier years when EBV DNA testing was not routinely performed (**Supplementary Table 8**). The significantly shorter follow-up (2.94 vs. 5.31 years, P < 0.001) and lower event rates (OS: 8.9% vs. 27.3%, *P* < 0.001) in the EBV-available group indicate that EBV DNA testing was gradually introduced at our center.

We explored the independent prognostic value of EBV DNA. In multivariate Cox models adjusting for Age, T stage, LDH, and C2, EBV DNA was not independently prognostic for any endpoint (**Supplementary Table 9**). To quantify whether incorporating EBV DNA changes the predictive performance of the nomogram, we compared three models: Model A [Original nomogram (Age + T stage + LDH + C2), n = 267], Model B [Original nomogram (same 4 variables), restricted to n = 190 (EBV-available subset)], and Model C [(5 variables including EBV DNA), n = 190]. The incremental C-index was +0.0067 (for OS) (**Supplementary Table 10**). This demonstrates that adding EBV DNA to the nomogram does not yield a meaningful improvement in discriminative ability.

To quantify the maximum potential bias from missing EBV DNA data, we performed extreme-scenario analyses on the full cohort (n = 267) under two assumptions: Worst case [All 77 missing EBV DNA values are assumed “detectable” (worst prognosis)] and Best case [All 77 missing EBV DNA values are assumed “undetectable” (best prognosis)]. The C-index spreads between worst-case and best-case scenarios were remarkably narrow (OS: 0.020; **Supplementary Table 11**). This indicates that even under the most extreme assumptions about missing EBV DNA values, the nomogram’s predictive performance remains stable, and the bias introduced by missing EBV DNA data is negligible.

## 4 Discussion

By means of a retrospective study, we identified C2 as an independent prognostic factor in LA-NPC. The nomogram combining C2 with conventional clinicopathological variables was constructed for estimating individual survival probability. The high-risk cohort experienced significantly worse outcomes compared to their low-risk counterparts. Notably, when high-risk individuals received IC followed by RT/CCRT, both OS and LRRFS were significantly prolonged relative to those treated with RT/CCRT alone. These results demonstrate that the constructed nomogram exhibits robust predictive performance and enables the identification of patients who benefit from IC.

The widely used TNM staging system serves as the cornerstone for therapeutic decision-making by delineating the anatomic extent of disease (21). Nevertheless, the prognosis of NPC patients is highly heterogeneous, even among those diagnosed at the same clinical stage (22, 23). The staging system fails to account for critical biological determinants of prognosis, such as intratumoral heterogeneity and variations in host systemic function. This prognostic uncertainty has fueled the search for reliable biomarkers and innovative assessment tools to enable more precise risk stratification and personalized treatment strategies. Recent advances highlight the significant prognostic value of various parameters beyond the TNM stage. These include radiomics analyses (24), circulating cell-free EBV DNA (25), and nutritional and inflammatory status (26). Accurate survival prediction therefore hinges on integrating these accessible biomarkers into practical decision-making tools.

Recently, considerable effort has been devoted to elucidating the association of nutritional and immune-inflammatory indicators with tumorigenesis, proliferation, distant metastasis, and prognosis (27, 28). Accumulating evidence has established that neutrophils and platelets critically regulate inflammation-mediated carcinogenesis, malignant progression, and clinical outcomes (29). In contrast, lymphocytes are essential for maintaining immune homeostasis and exerting anti-tumor effects against tumor proliferation and metastasis (30). Consequently, lymphocytopenia is frequently associated with diminished therapeutic efficacy and inferior clinical prognosis (31, 32). The prognostic relevance of various immune-inflammatory indices, such as PLR, NLR, and MLR, has been established across multiple cancer types (33, 34). Common nutritional indicators, including NRI, PNI, and albumin, have been widely utilized to evaluate the nutritional status and metabolic capacity (35–37). Both NRI and PNI primarily integrate serum albumin levels, lymphocyte counts, and body weight, with low levels frequently associated with adverse clinical outcomes. Previous studies have demonstrated that NPC patients with decreased NRI or PNI levels exhibit unfavorable survival prognosis (38–40).

However, previous studies typically focus on isolated biomarkers, which may fail to capture the complex, multidimensional interplay between the host’s nutritional status and systemic immune-inflammatory response (29, 41). Such univariate approaches often overlook the shared variance and potential collinearity among these indicators, potentially leading to incomplete or biased prognostic assessments. By applying PCA, we construct a composite index that synthesizes the collective contribution of both nutritional and immune-inflammatory factors. This multidimensional approach not only reduces data dimensionality but also more accurately reflects the host’s overall physiological state. Consequently, our method provides a more robust and holistic prognostic tool, potentially enhancing risk stratification and offering a more comprehensive perspective than traditional single-indicator analyses.

In this study, we chose C2 over C1 as the key prognostic indicator primarily for the following reasons based on statistical and biological evidence. Firstly, although C1 captured a larger proportion of total variance than C2, variance explained in PCA does not inherently equate to prognostic value. PCA is an unsupervised dimensionality-reduction technique that maximizes total variance without reference to clinical outcomes. Secondly, in our data, C1 was not significant in univariate analysis for any endpoint. We further confirmed that C1 remains non-significant (all *P* > 0.05) even when forced into the multivariate model, while C2’s prognostic significance is preserved. Thirdly, the inflammatory markers that dominate C1 (SII, NLR, PLR, MLR, SIRI) are acute-phase reactants that fluctuate with concurrent infections, treatment response, tumor necrosis, and corticosteroid use during chemoradiotherapy (33, 34). These fluctuations make the baseline inflammatory profile a less stable long-term prognostic indicator after adjustment for clinical variables (42). Moreover, C2 is primarily loaded on ALB, PNI, and NRI, markers of the host’s chronic nutritional reserve and metabolic state. Unlike inflammatory markers, serum albumin has a long half-life (21 days) and reflects sustained protein-energy status rather than acute-phase fluctuations.

The comprehensive nutritional status of patients requires multidimensional assessment through composite indicators. In this study, patients with elevated C2 levels exhibited a more favorable prognosis, and the potential underlying mechanisms may be as follows. First, patients with malnutrition or poor nutritional status typically present with decreased lymphocyte counts and hypoalbuminemia, often accompanied by compromised immune function, leading to diminished anti-tumor capacity and increased susceptibility to post-treatment infections (36). Conversely, patients with a higher C2 tend to maintain more robust immune competence, which may contribute to enhanced anti-tumor surveillance and reduced susceptibility to infections. Additionally, patients with suboptimal nutritional status may exhibit impaired repair capacity for radiation-induced injury, thereby exacerbating radiation-related adverse effects such as oral mucositis and radiation dermatitis (43). Therefore, the incorporation of C2 into the prognostic model facilitates the formulation of individualized treatment regimens.

This study provides novel insights into the dynamic prognostic value of C2 throughout chemoradiotherapy. C2 was measured at three time points, with no systematic drift observed across treatment phases (all paired t-tests P > 0.5). Patients were classified into four trajectory groups based on C2 status transitions. Those with persistently high C2 demonstrated the best prognosis, while those with persistently low C2 had the worst. These findings underscore the importance of longitudinal nutritional monitoring in identifying high-risk patients who may benefit from timely intervention. In multivariate analysis incorporating all three time points, pre-C2 retained independent prognostic significance for OS, whereas pre-RT C2 did not. This indicates that baseline nutritional status at admission is the dominant prognostic determinant, and pre-RT reassessment does not independently refine risk stratification. Clinically, this implies that nutritional intervention for low-C2 patients should be initiated immediately upon admission without awaiting pre-RT confirmation, as delaying intervention would subject at-risk patients to weeks of uncontrolled nutritional deterioration, during which treatment-related mucositis and dysphagia may progressively compromise oral intake. For these patients, systematic nutritional support, including dietary counseling, oral nutritional supplements (ONS), and prophylactic percutaneous endoscopic gastrostomy (PEG) when severe toxicities are anticipated, should be implemented promptly (44). Immunonutrition supplementation, including glutamine and omega-3 polyunsaturated fatty acids, may reduce the severity of radiation-induced mucositis and help maintain body weight during treatment (45, 46). Nutritional support should be managed within a multidisciplinary team framework, with dynamic adjustment based on treatment-related toxicities and weekly nutritional assessment, and continued for at least 3 months post-treatment. Furthermore, end-RT C2 independently predicted OS and is best utilized as a post-treatment surveillance tool. Unlike intra-treatment measurements subject to acute treatment-related fluctuations, end-RT C2 reflects the patient’s nutritional status after the completion of chemoradiotherapy and may identify patients at persistent risk who require extended nutritional follow-up and rehabilitation.

This study further revealed that survival outcomes varied across treatment regimens among patients stratified by nomogram-based risk scores. IC combined with CCRT significantly enhances OS and LRRFS only in the high-risk population, with no such benefit observed in the low-risk subgroup. Several biological and clinical rationales may account for this differential effect. Firstly, the disparity in treatment benefit is likely attributable to differences in tumor burden and biological aggressiveness. High-risk patients, typically characterized by advanced T or N categories, often present with bulky, hypoxic, and highly proliferative tumors. In these patients, IC serves a crucial cytoreductive function. By reducing gross tumor volume prior to CCRT, IC enhances oxygenation, improves drug delivery to residual disease, and minimizes the occurrence of relapse or metastasis (47, 48). In contrast, low-risk patients generally possess a lower baseline tumor burden. For these patients, the standard CCRT regimen may already be sufficient to eradicate micrometastases and local disease, leaving little residual tumor burden for IC to act upon. Secondly, the lack of benefit in the low-risk group could be partially attributed to overtreatment. In these patients, the added toxicity of IC, including hematologic, gastrointestinal, and infectious complications, may lead to increased rates of radiotherapy delays, unplanned interruptions of the definitive CCRT, or dose reductions (49, 50). Such compromised compliance can negate any potential therapeutic gain from the intensified regimen, ultimately resulting in comparable survival outcomes to those achieved with standard CCRT alone. Thirdly, low C2 status is one of the risk factors for high-risk patients. Patients with low C2 scores exhibit compromised nutritional reserves and systemic inflammation, which together create a malnutrition-inflammation-cancer vicious cycle. IC-mediated tumor cytoreduction reduces the tumor-driven inflammatory burden, thereby partially breaking the malnutrition-inflammation cycle before CCRT initiation (51, 52). Tumor cytoreduction alleviates tumor-induced immunosuppression, potentially restoring lymphocyte-mediated immune surveillance in patients whose baseline cellular immunity is already compromised by poor nutritional status (17, 33). Moreover, by reducing tumor volume before CCRT, IC may decrease the required radiation dose to adjacent healthy tissues and lessen cumulative treatment toxicity, which is particularly critical for patients with limited nutritional reserves who have reduced tolerance to CCRT-related mucositis and dysphagia (53, 54). In contrast, low-risk patients (high-nutritional reserve) possess adequate nutritional reserves and intact immune function, resulting in a narrower therapeutic window for additional IC benefit, as their baseline capacity to tolerate CCRT and mount an anti-tumor immune response is already sufficient (55).

Although EBV DNA plays a pivotal role in NPC pathogenesis and is a well-established prognostic biomarker (56), this study did not incorporate EBV DNA load as a nomogram parameter. At our center, the EBV DNA detection technique was not yet fully mature in earlier years, with limitations related to laboratory procedures, assay kits, and equipment (25). Moreover, EBV DNA data were incomplete in our cohort, with 77 of 267 patients (28.8%) lacking measurements. To quantify the potential bias introduced by these missing data and to evaluate whether incorporating EBV DNA would alter the nomogram’s predictive performance, we conducted a comprehensive sensitivity analysis. In multivariate Cox models adjusting for Age, T stage, LDH, and C2, EBV DNA was not an independent prognostic factor for any endpoint. The incremental C-index was +0.0067 (for OS), well below the minimal clinically important difference of 0.05 commonly used for C-index comparisons. Extreme-scenario analyses (worst-case and best-case imputation of missing values) yielded C-index spreads of only 0.020, confirming that the missing EBV DNA data do not materially bias the nomogram’s performance. Together, these findings demonstrate that the nomogram maintains robust predictive performance without EBV DNA, and its inclusion would not meaningfully improve discriminative ability.

A prognostic model combining C2 levels with additional essential clinical variables was developed, outperforming single biomarkers in predictive accuracy. It helps overcome limitations inherent in the conventional TNM staging system and offers a clinically useful tool for guiding treatment selection. Another strength of this study is the dynamic monitoring of C2 throughout chemoradiotherapy, which can support the clinical utility of C2 assessments as a real-time prognostic tracker during treatment.

Nevertheless, this research is subject to certain limitations. Firstly, this study was conducted at a single center with a retrospective design, which inherently introduces potential selection bias due to non-random patterns and institutional case-mix specificity, and limits the generalizability of the findings to other healthcare settings. To mitigate this bias, we employed several methodological safeguards: consecutive patient enrollment to minimize selection non-randomness, strictly standardized inclusion and exclusion criteria applied uniformly across the study period, comprehensive multivariable Cox regression adjusting for established prognostic confounders, and sensitivity analyses comparing models with and without EBV DNA to quantify the impact of missing data. However, we recognize that these measures cannot fully eliminate residual confounding from unmeasured variables. Secondly, the predictive performance of the proposed nomogram lacks external validation across multicenter cohorts to establish its generalizability. Although we performed bootstrap internal validation with 1,000 iterations and demonstrated acceptable bias-corrected discrimination with minimal optimism, the current study lacks external validation using an independent dataset from different institutions. Bootstrap resampling, while providing a robust internal validation framework, cannot fully assess model transportability across diverse clinical settings and patient populations. Future studies should prioritize multicenter prospective external validation across diverse geographic regions and treatment centers to verify the robustness, transportability, and generalizability of the nomogram before its widespread clinical adoption. Furthermore, integration of nomogram-derived risk scores into prospective randomized trials would provide stronger evidence for its clinical utility in risk-adapted treatment stratification for LA-NPC patients. Additionally, the association between nutritional enhancement therapy and prognosis in low-C2 patients was not further investigated in this study. Future prospective studies are needed to determine whether targeted nutritional strategies, stratified by PCA-derived immune-nutritional status, can improve survival outcomes in LA-NPC patients with poor baseline nutritional status.

## 5 Conclusions

A nomogram was developed by integrating C2 with additional clinical variables to facilitate prognostic prediction. The model serves as an effective tool for personalized risk assessment and treatment selection in LA-NPC patients.

## Data Availability

The data used and/or analyzed during the current study available from the corresponding author on reasonable request.
