# Supplementary material for: Prognostic significance of immune-inflammatory and nutritional indicators in locoregionally advanced nasopharyngeal carcinoma
Source: Front Immunol. 2026 Aug 31;17:1909926. doi: 10.3389/fimmu.2026.1909926 (PMC13572224; doi:10.3389/fimmu.2026.1909926)
Supplement: Supplementary file 1 [file DataSheet1.pdf]

1. Supplementary materials and methods

The formulae of the immune-inflammatory and nutritional indicators:

NLR (neutrophil-to-lymphocyte ratio) = neutrophil count (10^9/L)/lymphocyte count (10^9/L);

PLR (platelet-to-lymphocyte ratio) = platelet count (10^9/L)/lymphocyte count (10^9/L);

MLR (monocyte-to-lymphocyte ratio) = monocyte count (10^9/L)/lymphocyte count (10^9/L);

SII (systemic immune-inflammatory index) = (platelet count (10^9/L) × neutrophil count (10^9/L) × 10^{-9})/lymphocyte count (10^9/L);

SIRI (systemic inflammation response index) = (neutrophil count (10^9/L) × monocyte count (10^9/L) × 10^{-9})/lymphocyte count (10^9/L);

PNI (prognostic nutritional index) = serum albumin (g/L) + 5 × 10^{-9} × lymphocyte count (10^9/L);

NRI = [1.519 × serum albumin (g/L)] + [41.7 × (current body weight / ideal body weight)];

Ideal body weight (kg) = 22.5 × height $(m)^2$.

Treatment

Induction chemotherapy (IC), administered at three-week intervals, was applied in over 85% of patients. The IC regimens included GP (gemcitabine 1 g/m^2, days 1 and 8; cisplatin 80-100 mg/m^2 over 3 days) and TP (paclitaxel 175 mg/m^2 or docetaxel 60-75 mg/m^2, day 1; cisplatin 80-100 mg/m^2 over 3 days). For patients ineligible for cisplatin, equivalent doses of carboplatin or nedaplatin were substituted based on renal function and tolerance.

Definitive intensity-modulated radiotherapy (IMRT) was administered using 6-MV photons. The gross tumor volumes of the primary tumor (GTV-T) and metastatic lymph nodes (GTV-N) were delineated by integrating diagnostic imaging, clinical examination, and endoscopic findings. Corresponding clinical target volumes (CTV) were defined as follows: CTV1 covered regions at high risk for microscopic spread, and CTV2 encompassed areas of potential subclinical involvement. The prescribed radiation doses were 66-76 Gy to GTV-T and GTV-N. Critical organs at risk (e.g., brainstem, spinal cord, parotid glands, and optic nerves) were contoured for dose optimization, with all constraints strictly met.

2. Supplementary Tables

Table S1. Eigenvalues and percentage of total variance explained by each component.

Component	Initial Eigenvalues			Extraction Sum of Squared Loadings		
	Total	% of Variance	Cumulative %	Total	% of Variance	Cumulative %
1	4.178	52.231	52.231	4.178	52.231	52.231
2	2.175	27.189	79.420	2.175	27.189	79.420
3	0.746	9.330	88.750			
4	0.353	4.418	93.168			
5	0.318	3.973	97.142			
6	0.167	2.088	99.230			
7	0.045	0.567	99.797			
8	0.016	0.203	100.000			

Table S2. Kaiser-Meyer-Olkin (KMO) Measure of Sampling Adequacy.

Variable	KMO
NLR	0.739
PLR	0.451
MLR	0.530
SII	0.520
SIRI	0.499
PNI	0.591
NRI	0.881
ALB	0.506

Table S3. Bartlett's Test of Sphericity.

**Statistic**	**Value**
Chi-square (χ^2)	2434.50
Degrees of freedom (df)	28
P-value	$P < 0.001$

Table S4. Univariate analysis for the nasopharyngeal carcinoma patients.

	OS		PFS		DMFS		LRRFS	
Variables	HR (95%CI)	P	HR (95%CI)	P	HR (95%CI)	P	HR (95%CI)	P
Age (years)	1.04 (1.01-1.07)	0.005**	1.01 (0.99-1.04)	0.212	1.02 (1.00-1.05)	0.104	1.03 (1.01-1.06)	0.021*
Gender								
Female	1.00 (Reference)		1.00 (Reference)		1.00 (Reference)		1.00 (Reference)	
Male	1.77 (0.83-3.75)	0.138	1.55 (0.85-2.84)	0.152	1.63 (0.83-3.21)	0.158	1.51 (0.80-2.85)	0.205
T category								
T1/T2	1.00 (Reference)		1.00 (Reference)		1.00 (Reference)		1.00 (Reference)	
T3/T4	2.38 (0.92-6.13)	0.074	2.01 (0.95-4.27)	0.068	2.15 (0.91-5.09)	0.082	2.14 (0.96-4.78)	0.064
N category								
N0/N1	1.00 (Reference)		1.00 (Reference)		1.00 (Reference)		1.00 (Reference)	
N2/N3	1.12 (0.58-2.17)	0.737	1.51 (0.86-2.63)	0.150	1.61 (0.86-3.00)	0.138	1.14 (0.64-2.04)	0.651
Overall stage								
I-II	1.00 (Reference)		1.00 (Reference)		1.00 (Reference)		1.00 (Reference)	
III-IVa	1.70 (0.90-3.23)	0.102	1.88 (1.12-3.16)	0.017*	1.69 (0.95-3.01)	0.072	1.81 (1.04-3.17)	0.037*
IC								
No	1.00 (Reference)		1.00 (Reference)		1.00 (Reference)		1.00 (Reference)	
Yes	0.59 (0.29-1.22)	0.157	0.68 (0.36-1.29)	0.242	0.68 (0.34-1.37)	0.282	0.63 (0.33-1.21)	0.162

CCRT								
No	1.00 (Reference)		1.00 (Reference)		1.00 (Reference)		1.00 (Reference)	
Yes	0.74 (0.26-2.08)	0.567	0.80 (0.34-1.87)	0.612	0.98 (0.35-2.74)	0.975	0.65 (0.28-1.53)	0.323
C1								
< -0.372	1.00 (Reference)		1.00 (Reference)		1.00 (Reference)		1.00 (Reference)	
≥ -0.372	0.71 (0.37-1.36)	0.305	1.02 (0.61-1.71)	0.940	1.06 (0.60-1.87)	0.853	0.77 (0.44-1.35)	0.360
C2								
< -0.242	1.00 (Reference)		1.00 (Reference)		1.00 (Reference)		1.00 (Reference)	
≥ -0.242	0.35 (0.17-0.71)	0.003**	0.44 (0.26-0.75)	0.003**	0.35 (0.19-0.64)	<0.001***	0.46 (0.26-0.82)	0.008**
LDH	1.01 (1.01-1.01)	0.021*	1.01 (1.01-1.01)	0.012*	1.01 (1.01-1.01)	0.002**	1.00 (1.00-1.01)	0.053

Abbreviations: OS = overall survival; PFS = progression-free survival; DMFS = distant metastasis-free survival; LRRFS = locoregional relapse-free survival; IC = induction chemotherapy; CCRT = concurrent chemoradiotherapy; C1 = component 1; C2 = component 2; LDH = lactate dehydrogenase; *$P < 0.05$; **$P < 0.01$; ***$P < 0.001$.

Table S5. C2 distribution and cutoff-based classification at three time points.

Time Point	N	Missing	Median	High group (⩾ −0.242)	Low group (< −0.242)
Pre-C2	267	0	0.0292	157 (58.8%)	110 (41.2%)
Pre-RT C2	267	0	0.1434	164 (61.4%)	103 (38.6%)
End-RT C2	256	11	0.0838	150 (58.6%)	106 (41.4%)

Table S6. Paired C2 changes across treatment phases.

Transition	N (paired)	Paired t-test	Increased	Decreased
Pre-C2 → Pre-RT C2	267	t=0.00, P = 1.0000	143 (53.6%)	124 (46.4%)
Pre-RT C2 → End-RT C2	256	t=-0.58, P = 0.5627	129 (50.4%)	127 (49.6%)
Pre-C2 → End-RT C2	256	t=-0.65, P = 0.5143	122 (47.7%)	134 (52.3%)

Table S7. Univariate analysis and multivariate analysis for OS.

	Univariate analysis		Multivariate analysis	
Variables	HR (95%CI)	P	HR (95%CI)	P
Pre-C2				
< -0.242	1.00 (Reference)		1.00 (Reference)	
≥ -0.242	0.35 (0.17-0.71)	0.003**	0.42 (0.19-0.94)	0.034*
Pre-RT C2				
< -0.242	1.00 (Reference)		1.00 (Reference)	
≥ -0.242	0.55 (0.29-1.06)	0.075	1.28 (0.60-2.74)	0.530
End-RT C2				
< -0.242	1.00 (Reference)		1.00 (Reference)	
≥ -0.242	0.34 (0.16-0.71)	0.004**	0.38 (0.18-0.81)	0.013*

Abbreviations: OS = overall survival; C2 = component 2; *P < 0.05; **P < 0.01.

Table S8. Distribution of Plasma EBV DNA Status.

EBV DNA Status	N	Percentage (%)	OS Events	OS Rate (%)
Missing	77	28.8	21	27.3
Undetectable (Negative)	103	38.6	3	2.9
Detectable (Positive)	87	32.6	14	16.1
Total	267	100.0	38	14.2

Table S9. Multivariate Cox regression with EBV DNA (n = 190).

	OS		PFS		DMFS		LRRFS	
Variables	HR (95%CI)	P	HR (95%CI)	P	HR (95%CI)	P	HR (95%CI)	P
Age	1.018 (0.974-1.064)	0.428	0.972 (0.941-1.005)	0.092	0.976 (0.94-1.014)	0.207	0.998 (0.963-1.034)	0.893
T category								
T1/T2	1.00 (Reference)		1.00 (Reference)		1.00 (Reference)		1.00 (Reference)	
T3/T4	2.119 (0.598-7.502)	0.244	1.403 (0.553-3.560)	0.475	1.343 (0.474-3.803)	0.578	1.447 (0.509-4.109)	0.487
C2								
< -0.242	1.00 (Reference)		1.00 (Reference)		1.00 (Reference)		1.00 (Reference)	
≥ -0.242	0.496 (0.171-1.44)	0.197	0.412 (0.191-0.892)	0.024*	0.303 (0.123-0.749)	0.009**	0.682 (0.285-1.634)	0.390
LDH	1.010 (1.002-1.018)	0.012*	1.007 (1.002-1.012)	0.008**	1.009 (1.003-1.015)	0.001**	1.007 (1.000-1.014)	0.062
EBV DNA								
negative	1.00 (Reference)		1.00 (Reference)		1.00 (Reference)		1.00 (Reference)	
positive	2.503 (0.653-9.597)	0.180	1.332 (0.611-2.905)	0.471	2.015 (0.773-5.252)	0.151	1.329 (0.537-3.288)	0.538

Abbreviations: OS = overall survival; PFS = progression-free survival; DMFS = distant metastasis-free survival; LRRFS = locoregional relapse-free survival; C2 = component 2; LDH = lactate dehydrogenase; EBV = Epstein-Barr virus; *$P < 0.05$; **$P < 0.01$.

Table S10. C-index Comparison: Original vs. EBV-available Models.

Endpoint	Model A	Model B	Model C	Increment (C-B)
OS	0.7380	0.7230	0.7297	+0.0067

Model A [Original nomogram (Age + T stage + LDH + C2), n = 267], Model B [Original nomogram (same 4 variables), restricted to n = 190 (EBV-available subset)], and Model C [(5 variables including EBV DNA), n = 190].

Table S11. Extreme-Scenario Sensitivity Analysis.

Endpoint	Worst Case C-index	Best Case C-index	Spread (|Worst - Best|)
OS	0.7569	0.7369	0.0200

Worst case = all missing EBV DNA treated as “detectable”. Best case = all missing EBV DNA treated as “undetectable”.

3. Supplementary Figures

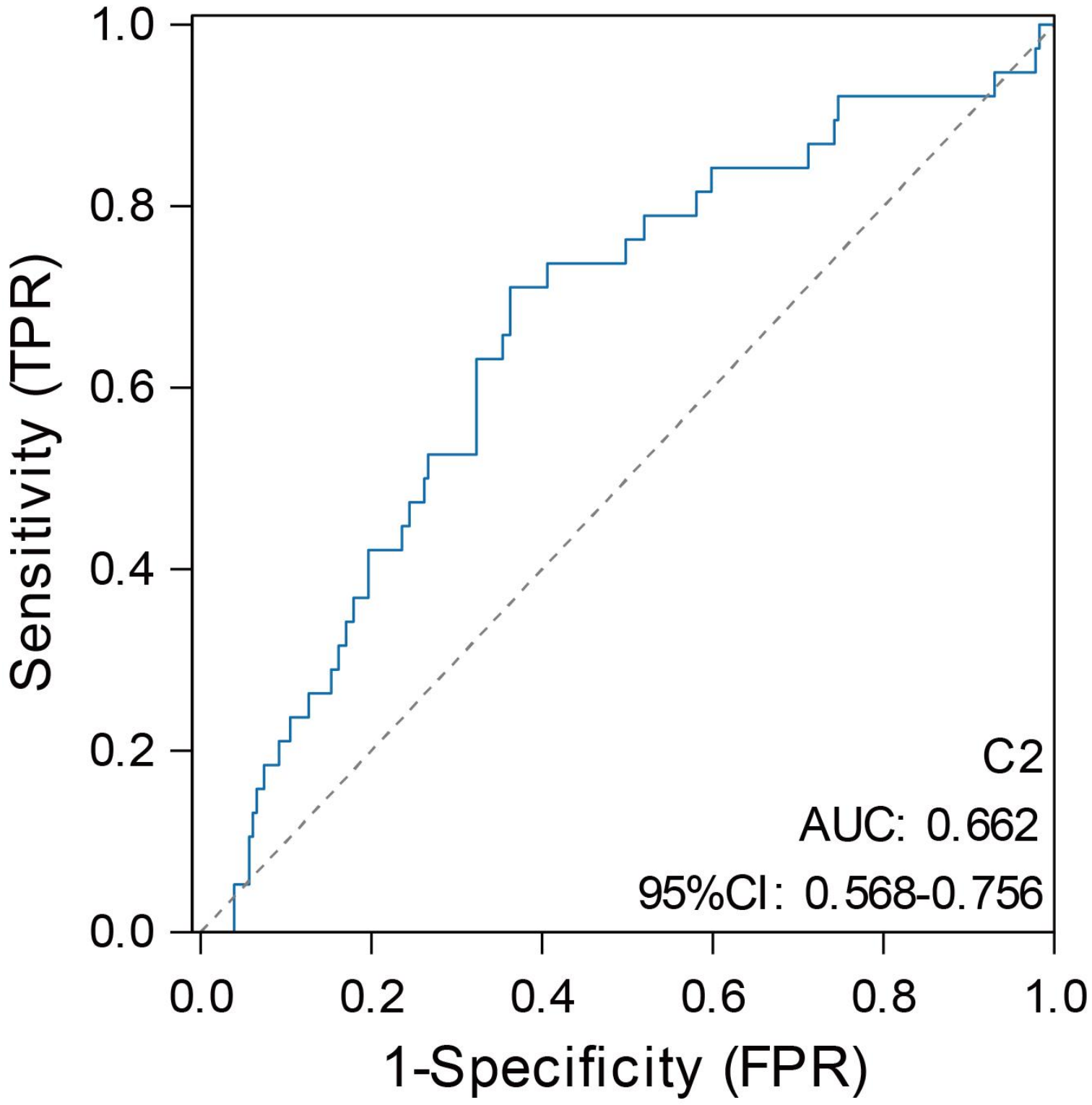

Figure S1. The receiver operating characteristic curve of C2 for predicting OS.

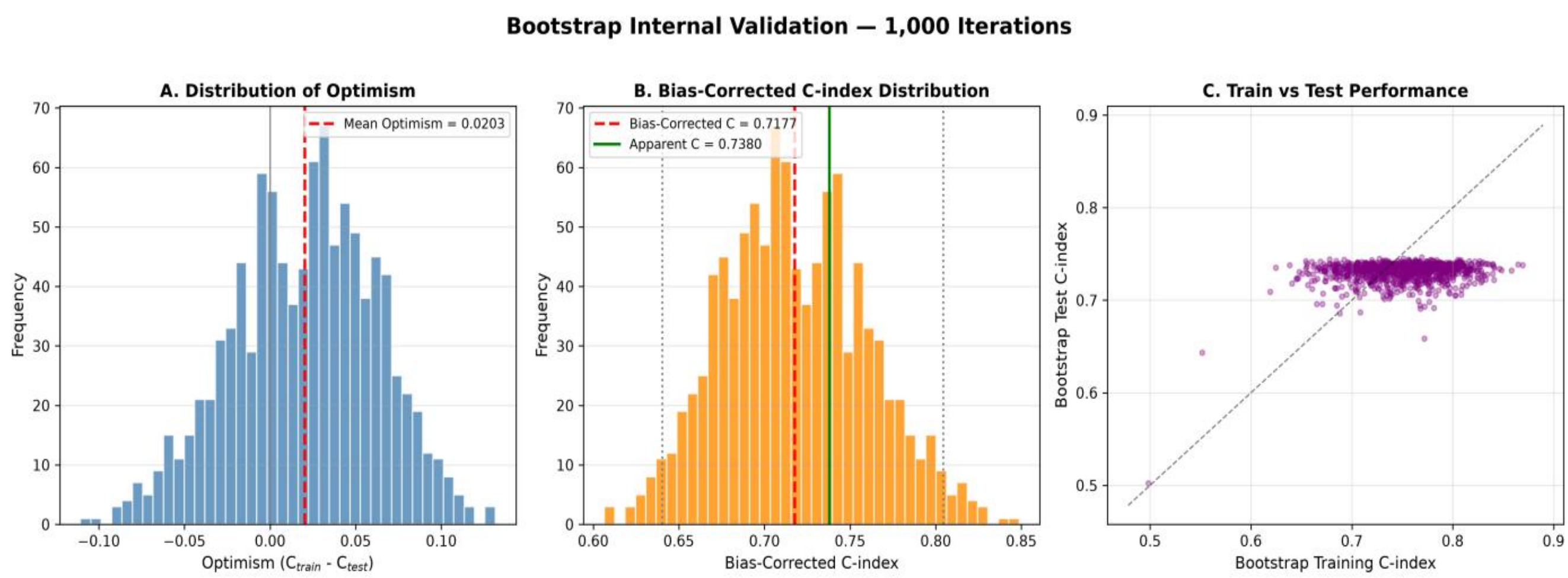

Figure S2. Bootstrap Internal Validation: (A) the distribution of optimism across 1,000 bootstrap replicates; (B) the distribution of bias-corrected C-index values; (C) a scatter plot comparing training versus test C-indices.

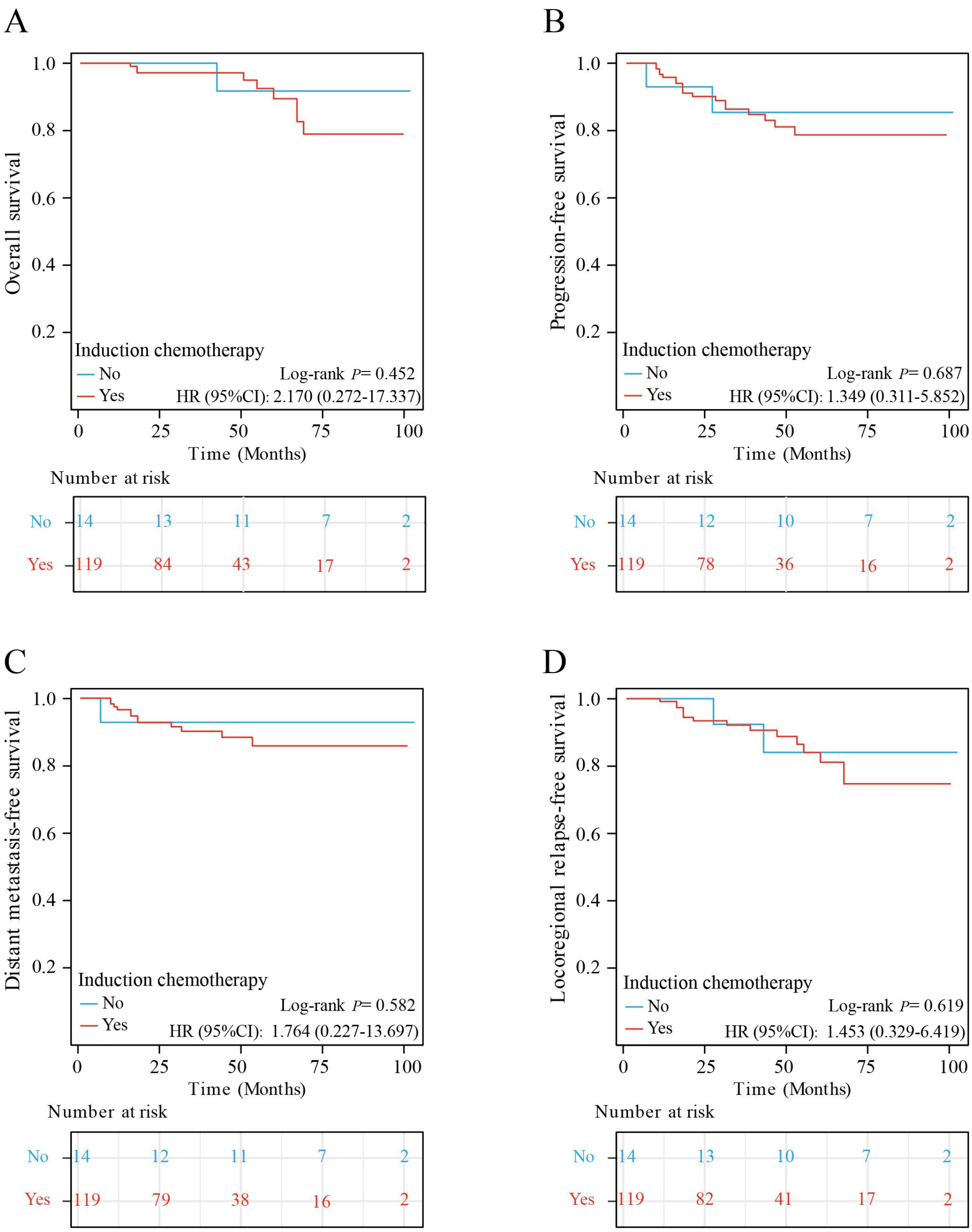

Figure S3. Kaplan-Meier survival curves of (A) OS, (B) PFS, (C) DMFS and (D) LRRFS for NPC patients receiving induction chemotherapy in the low-risk group.
